# Translation and validation of the German version of the Nursing Brand Image Scale (NBIS-P-G)

**DOI:** 10.1186/s12912-025-04025-5

**Published:** 2025-11-11

**Authors:** Clemens Koob, Bernd Reuschenbach, Judi Allyn Godsey

**Affiliations:** 1 Faculty of Healthcare and Nursing, Catholic University of Applied Sciences Munich, Preysingstraße 95, 81667 Munich, Germany; 2 Institute for the Brand Image of Nursing, Covington, KY 41011 USA

**Keywords:** Brand Image of Nursing, Translation, Validation, Cross cultural, Psychometric properties, Nursing Brand Image Scale, Germany, Public perceptions

## Abstract

**Background:**

The global nursing shortage significantly impacts healthcare systems, making nursing’s public image crucial for workforce sustainability. Public perceptions influence career choice, professional identity, and retention. Germany lacks a validated instrument to assess these perceptions. The Nursing Brand Image Scale (NBIS), rooted in brand theory, assesses perceptions via functional and symbolic associations. This study aimed to translate and validate the NBIS for the German public (NBIS-P-G).

**Methods:**

An online quota sample representative of the German population (*N* = 950; aged 16–65) was surveyed. Translation followed established guidelines. Structural validity was assessed via exploratory (EFA; *N* = 420) and confirmatory factor analyses (CFA; *N* = 530). Convergent and discriminant validity, internal consistency, and concurrent validity (correlations with brand attitudes, occupational prestige, career recommendation, political solidarity) were examined.

**Results:**

A four-factor solution emerged (Professional Competence and Expertise; Patient-Centered Care and Compassion; Leadership and Influence; Professional Identity Challenges), differing from the original seven-factor NBIS. CFA indicated acceptable fit for the four-factor structure (RMSEA = 0.045, SRMR = 0.054, CFI = 0.942). Internal consistency was strong (α, ω = 0.74–0.95). Convergent, discriminant, and concurrent validity were generally supported.

**Conclusions:**

The NBIS-P-G demonstrates sound psychometric properties and provides a basis for monitoring public perceptions and evaluating branding initiatives. Further refinement and measurement invariance studies are recommended.

## Background

Health workforce and human resource planning are recognized as priorities for health systems globally. The shortage of nursing personnel presents a significant threat to healthcare delivery. The International Council of Nurses has characterized this situation as a “global health workforce crisis” [1], with current estimates suggesting a deficit of 6 million nurses [2] and projections of a global shortfall of up to 30 million nurses in the future [3].

Career choice decisions in nursing are significantly influenced by the profession’s public image [4, 5]. While comparative analyses examining the relative impact of image versus other career decision factors are limited, numerous studies demonstrate significant associations between perceived professional prestige, role clarity, and societal value with career choices – particularly among young people [6–12]. When nursing is associated with low autonomy, limited career development, or traditional gender roles, it may be seen as less attractive compared to other healthcare or academic career paths. Conversely, a strong, modern brand image of nursing – highlighting complexity, responsibility, and professional scope – can support efforts to recruit the next generation into the workforce [5, 13].

The public image of nursing also has profound implications for professional identity and retention. Nurses’ perceptions of how they are viewed by society can affect professional identity, self-concept, and professional pride [5]. A negative public image may lead to a diminished self-concept [14], while a well-regarded image can reinforce professional identity, enhance resilience, and foster a sense of legitimacy and recognition [15]. A poor image has been identified as a push factor leading nurses to leave the profession, while social recognition and a positive image can serve as motivators to stay [16].

The public image of nursing is also a key consideration in the global context of nursing migration [1]. Many countries facing nursing shortages are seeking to recruit nurses internationally, while source countries are introducing policies to reduce outward migration to mitigate its potential detrimental effects on the healthcare system and beyond [17–19]. In this context, the image of nursing acts as both a push and pull factor, alongside economic, educational, historical, legal, political, and sociocultural variables [20–22].

In addition to these aspects, recent reviews have identified a wide range of further outcomes linked to the public image of nursing, from effects on nurses’ job performance, job satisfaction, work-life-quality, and remuneration to organizational and public-level consequences such as interprofessional collaboration, care quality, and overall health outcomes [5, 13]. Procedural justice theory helps explain how public perceptions translate into health outcomes by influencing patient-health professional interactions. When health professionals – and the group as a whole [23] – are perceived as procedurally just (offering patients voice and using transparent, fact-based decision-making procedures) and interpersonally fair (treating patients with dignity and respect, signaling concern for their well-being) [24], patients are more likely to voluntarily accept and comply with guidance [23], in part because such encounters foster trust. Consistent with this pathway, trust in nurses has been identified as a key factor associated with higher medication adherence among cardiovascular patients [25]. In this view, a positive brand image of nursing may also strengthen the effectiveness of care delivery.

Given its relevance, there is a pressing need for further research into the image of nursing, including cross-cultural comparisons [5, 13, 26]. However, such efforts require internationally comparable, validated instruments for determining image. Several instruments have been developed to assess perceptions of nursing, but most existing tools are tailored toward nurses’ self-image, rather than public perceptions [27]. Although theoretically related, these are distinct constructs [26, 28], and studies show divergent patterns between the two perspectives [29, 30].

In the German context, this gap is particularly pronounced. No validated instrument currently exists that systematically captures the public image of the nursing profession. While some studies have explored public perceptions in Germany, these often rely on secondary data [31, 32], media analyses [33], or non-standardized surveys in subgroups such as adolescents [34]. These studies typically use ad hoc items lacking theoretical and psychometric rigor. The absence of a validated German-language instrument impedes research and targeted practical interventions, limiting the ability to monitor trends, evaluate image-shaping campaigns and policy initiatives, or conduct international comparisons.

Among existing instruments, the Nursing Brand Image Scale (NBIS) [35] stands out as particularly promising for adaptation to the German context. The brand image of nursing refers to the collective, socially constructed perception of the nursing profession held by the public, media, and nurses themselves. It encompasses how nurses are portrayed, understood, and valued in terms of their roles, competencies, leadership, and contributions to health and society [35]. This image is shaped by historical narratives, professional identity, media representations, and strategic communication by nurses, nursing organizations, and other actors [5, 13]. A strong and unified brand image enhances professional recognition, policy influence, and recruitment into the profession [36]. The NBIS can be used with both nurses and the public to assess perceptions of the nursing profession and the (mis)alignment between how nurses see themselves and how they are perceived by others [35]. A recent review recommended the NBIS for its comprehensiveness, content validity, and strong theoretical grounding [27]. Uniquely, the NBIS is rooted in brand image theory from marketing, conceptualizing nursing as a professional brand [35, 37]. Although the concept of nursing image is still debated [5, 13], the NBIS is grounded in a clear theoretical framework that defines nursing brand image as functional, symbolic, and experiential associations stored in memory [38, 39] – i.e., the network of positive and negative mental associations people form about nursing [40]. These associations can be evaluated in terms of their favorability, strength, uniqueness, relevance, accuracy, and consistency over time [29, 35, 38, 39]. This brand image perspective enables a multidimensional analysis of public perceptions of nursing – encompassing not only evaluations of the profession’s competence and functional value, but also its symbolic meaning and cultural significance within broader societal narratives. In line with recent reviews highlighting both intrinsic (e.g., knowledge, values) and extrinsic (e.g., appearance, behavior) aspects of the nursing image [5, 13], the NBIS captures a broad range of attributes across factors [35]. Its foundation in marketing theory allows for alignment with established research in brand image measurement [40, 41], strategy, and communication [42], as well as occupational branding [43–45]. This perspective can guide the development and evaluation of initiatives to enhance nursing’s public image.

Originally developed to assess nurses’ self-perceptions, the NBIS contains 42 descriptors (words and phrases) rated on a 10-point scale [35]. The original study with U.S. registered nurses demonstrated good internal consistency and a seven-factor structure [35]. A Chinese version has also shown strong psychometric properties in a national nurse sample (content validity, structural validity, construct validity, internal consistency, test-retest reliability, responsiveness, no floor/ceiling effect) [46]. More recently, a slightly adapted version of the NBIS has been used to assess public perceptions of nursing in the U.S. and China [26], again showing adequate psychometric properties.

Building on this foundation, the current study undertakes the first systematic translation, adaptation, and validation of the NBIS for use with the German general population.

## Methods

### Study design

The study utilized a two-phase design. The first phase involved translation of the English version of the original NBIS using established translation guidelines with permission granted from the author (A). The second phase encompasses validation of the German version (B).

### (A) Translation and cultural adaption of the NBIS

The NBIS includes 42 descriptors potentially characteristic of the nursing profession [35]. In both the original U.S. scale development study [35] and the Chinese validation [46], the NBIS demonstrated a seven-factor structure, consisting of the following domains: Strong Interpersonal Skills, Expert Health-Care Providers and Partners, Valued By Society, Qualified Caregivers, Influential Leaders / Interprofessional Partners, Qualified for Advanced Nursing Practice, and Lack Authority / Professional Identity.

Translation and cultural adaption followed established guidelines [47–50] and comprised forward translation, synthesis, back translation, expert review, and pretesting.

#### Forward translation

Two independent forward translations of the original English scale into German were conducted using distinct AI applications based on different language models: ChatGPT-4.5 (T1) and Claude 3.7 Sonnet (T2) [48]. The translation prompt was engineered in line with established criteria for high-quality translation [49]. AI-based translation was deemed viable given the high genetic similarity between English and German, as prior research has shown that under such conditions, AI can produce survey translations approaching human-level quality [48]. Additionally, research suggests that AI and human translators exhibit complementary strengths in translation tasks [51]. To ensure rigor, the entire AI-assisted translation process was designed in accordance with current recommendations for using AI in cross-cultural questionnaire translation [48], including expert review and verification, as described below.

#### Synthesis

An expert committee harmonized the two forward translations to develop a single version (T12). The committee also addressed gender-inclusive language in the translation. Members of the committee included four German bilingual scholars – the principal investigator (CK), one of the co-authors (BR), a medical doctor and public health specialist (NS), and a management scholar (LH) – and an English native speaker (SL), fluent in German and familiar with German culture.

#### Back translation

The harmonized German version (T12) was independently back-translated into English by the same two AI models (ChatGPT-4.5 [B1], Claude 3.7 Sonnet [B2]), again utilizing a text prompt designed to maintain high-quality output.

#### Expert committee review

The expert committee reviewed all translated versions considering semantic, idiomatic, experiential, and conceptual equivalence. Discrepancies arising between the versions were initially resolved through discussions. ChatGPT-4.5 and Claude 3.7 Sonnet were also consulted for assistance during this phase. Unresolved questions were clarified in consultation with one of the originators of the instrument (Judi Allyn Godsey, JG).

#### Pretesting

Subsequently, the pre-final translated German version was pretested for comprehensibility with 41 students (bachelor’s and master’s level) from a university in Southern Germany, and resulting comments were included. The accuracy of the final translated German version was confirmed by JG as original NBIS developer.

The final translated German version of the NBIS for the public (NBIS-P-G), comprising 42 items, is presented in Table 1. This version served as the basis for the subsequent psychometric analyses.

Participants were asked to rate the extent that the items apply to the nursing profession on a 10-point Likert scale ranging from “1 = does not apply at all” to “10 = perfectly applies” as in the original version.

**Table 1:** Final translated German version of the NBIS for the public (NBIS-P-G; 42 items before validation)

Items	
1	eigenständig (selbständige Entscheidungsträger*innen)
2	einflussreich
3	mächtig/Entscheidungsträger*innen
4	evidenzbasierte Praxis (Pflege basierend auf wissenschaftlichen Erkenntnissen)
5	Führungspersonen
6	interprofessionell (arbeitet als Teil eines Teams von Fachleuten)
7	achtsam und umsichtig
8	Brückenbauer*innen in der Kommunikation (vermitteln zwischen Patient*innen, Angehörigen und Fachleuten, um Verständnis und Vertrauen zu fördern)
9	kompetent
10	interprofessionelle Partner*innen (arbeiten auf Augenhöhe mit Fachkräften aus Medizin, Therapie, Sozialarbeit und anderen Bereichen zusammen)
11	reflektiert/kritisch denkend
12	professionell
13	sachkundig/intelligent
14	ehrlich/Integrität
15	ethisch
16	Pflegewissenschaftler*innen (leiten und beteiligen sich an Forschung zur Verbesserung der Pflege)
17	ganzheitlicher Ansatz
18	höhere Bildungsabschlüsse (hochschulische Ausbildung)
19	umfassende Ausbildung
20	verlässlich/zuverlässig
21	fachkundig
22	Lehrpersonen/Ausbilder*innen^†^
23	talentiert/begabt
24	kompetent im Umgang mit Technik und digitalen Systemen
25	verbringt die meiste Zeit mit Patient*innen
26	genießt Vertrauen
27	empathisch
28	fürsorglich/mitfühlend
29	Fürsprecher*innen (setzen sich aktiv für die Rechte, Bedürfnisse und Anliegen von Patient*innen und des Pflegeberufs ein)
30	patientenorientiert
31	ärztliche Hilfskraft
32	aufgabenfixiert^†^
33	umsorgend/bemutternd
34	schwer von anderen Gesundheitsfachkräften zu unterscheiden
35	die Berufskleidung vermittelt keine Professionalität / keinen Status
36	untergeordnet/dienend
37	weiblich^†^
38	Gesundheitsdienstleister*innen^†^
39	Gesundheitsexpert*innen
40	unverzichtbare Mitglieder des Gesundheitsteams^†^
41	vielfältige berufliche Möglichkeiten^†^
42	von der Gesellschaft/vom Gesundheitswesen wertgeschätzt

Note: Items marked with ^†^ were excluded from the final version of the NBIS-P-G based on psychometric criteria. See Results for details

### (B) Validation of the instrument

Validation was conducted using an online survey. To assess validity, additional measurement instruments were incorporated. Data collection was realized using the web-based platform EFS Survey (version 24.2). Data were collected in summer 2025.

#### Participants

Individuals between 16 and 65 who lived in Germany were eligible to participate in the study. Participants were recruited from an online access panel, as widely done in research [52, 53], to facilitate data collection. Cint was chosen as provider, since they operate one of the largest online access panels in Germany with more than 4 million participants and adhere to a transparent sample process in terms of the ESOMAR framework for evaluating online sampling services [54]. Potential study participants were asked screening questions on age, gender, education, employment, and region of residence to verify inclusion criteria and allow stratification of the sample. Non-interlocking quotas [46] for the demographic criteria were applied to obtain a sample that mirrors the target population’s demographic composition.

All participants provided their informed consent before engaging in the study. The data were collected in accordance with the European Union’s General Data Protection Regulation. Furthermore, this study received ethical approval from the Interdisciplinary Research Ethics Committee at the Catholic University of Applied Sciences Munich (2024/N48-2).

#### Other measures

Since there are no standard measures of public nursing image that could be used as a reference to compare the validity of the NBIS-P-G [27], other established instruments with documented psychometric properties were used to assess concurrent validity. Pairwise correlations of the NBIS-P-G factors with brand attitudes, occupational prestige, career recommendation likelihood, and political solidarity were calculated.

*Brand attitudes* are considered to be part of brand equity [38, 39] and are defined as an “individual’s internal evaluation of the brand” [55]. Nursing profession brand attitudes thus refer to a relatively enduring, unidimensional summary evaluation of the nursing profession that presumably energizes behavior [56] (e.g., personal action tendencies relating to the profession such as recommending a career in nursing). We measured nursing profession brand attitudes employing the well-established and psychometrically validated Attitude toward the Brand Scale [56, 57] in its German version [58] consisting of five items. An example item was “unappealing / appealing”. The items had a seven-point semantic differential format with higher values reflecting more positive evaluations of the nursing profession brand.

*Occupational prestige* can be conceptualized as society’s perception of the desirability of an occupation [59]. We measured occupational prestige using the recently introduced and validated German occupational prestige scale (called BAS) [60, 61]. The prestige judgements of the nursing profession were collected via an end-verbalized scale ranging from 0 (“very low prestige”) to 10 (“very high prestige”).

Moreover, the *career recommendation likelihood*, i.e., the chance that an individual would recommend a career as a nursing professional to a close friend or family member was measured using the instrument developed by Abner et al. [62], which is grounded in established behavioral intention research [62] and includes a scale from 0% (indicating the least likelihood) to 100% (indicating the greatest likelihood). Participants could choose any number in between by sliding a bar to mark their response.

*Political solidarity* with nursing was assessed with the allyship and social change commitment subscales of the psychometrically validated Political Solidarity Measure (PSM) [63], each consisting of three items. Example items were “I feel a sense of solidarity with the nursing profession” (allyship) and “Policies negatively affecting the nursing profession should be changed” (social change commitment). The measure used a 7-point agreement scale with higher values signifying stronger solidarity.

Furthermore, *socio-demographic* background information was captured to verify the inclusion criteria and to describe the sample. These questions were formulated in accordance with established national surveys.

#### Bias and data quality

To alleviate data quality concerns related to the reliance on an online panel, we adhered to recommendations in the literature [53, 64]. In addition to the panel provider’s vetting mechanisms [65] and the aforementioned in-survey screener questions, respondents were asked at the beginning of the questionnaire to explicitly commit to answering carefully by clicking a corresponding on-screen option [66]; those who did not commit were excluded from the study. Furthermore, attentiveness checks were applied to ensure high-quality responses, excluding respondents failing these questions [67]. Additionally, the longstring index – defined as the maximum number of identical consecutive responses – was calculated for the NBIS-P-G scale. Participants with a string of consistent responses equal or greater than half the length of the total scale were considered as careless or insufficient effort responders [67] and were excluded. The potential for economic self-selection bias [52] was alleviated by controlling the socioeconomic composition of the sample, and by offering a fair but not overly appealing incentive for participation [64]. Mandatory answering was used to avoid missing data [68].

#### Sample size estimation

To derive the minimal sample size for the study, two perspectives were considered in addition to the general rule of thumb for CFA sample size advocated by Lambert and Newman (*N* ≈ 200 or greater) [69].

First, sample size considerations were guided by CFA criteria to ensure model convergence, unbiased chi-square estimates, and sufficient statistical power [70, 71]. Applying Koran’s heuristic [70] for the planned CFA model with f = 7 factors, at least p/f = 2 indicators per factor, and standardized factor loadings of a = 0.8 suggested a required sample of at least 655 participants.

Second, since pairwise correlation analyses were planned to assess concurrent validity, we determined the minimum sample size required to obtain sufficient statistical power to detect correlations between the measures of interest. Assuming an alpha level of α = 0.05 (two-tailed), an effect size of ρ = 0.30 – representing the threshold for low concurrent validity [72] – and a target power of 1-β = 0.80, an a-priori power analysis using G*Power (Version 3.1.9.6 for Mac) indicated that a minimum of 84 respondents would be required.

Considering both perspectives outlined above, we defined a minimum required sample size of 655 participants for this study (the maximum of the values resulting from the perspectives).

#### Statistical analyses

SPSS Statistics (version 29), R (version 4.3.2) and RStudio (version 2023.06.1 + 524) were used for all analyses. All analyses closely followed established guidelines for validating measures [69, 73].

##### Processing of the sample and data preparation

First, we verified that all respondents met the eligibility criteria, commitment and attention checks. Participants not meeting these criteria were removed from the sample. Furthermore, we excluded participants that failed the longstring check, with the longstring index computed using the careless package (Version 1.2.2). Since mandatory answering was used, no missing values were identified in the dataset, obviating the need for using methods for treating missing data such as listwise deletion or imputation procedures. Subsequently, the structure of the sample was described.

##### Structural validity

To examine the proposed seven-factor structure of the NBIS, a confirmatory factor analysis was conducted. The R packages lavaan (Version 0.6–19), semTools (Version 0.5-7) and psych (Version 2.3.12) were used for the analyses. Given multivariate non-normality (Mardia’s test: skewness = 274.78, *p* < 0.001; kurtosis = 2,744.24, *p* < 0.001), robust maximum likelihood estimation (MLR) was applied. Model fit was evaluated using standard indices and criteria [71, 74, 75]: RMSEA ≤ 0.05 (90% CI ≤ 0.05), SRMR ≤ 0.08, CFI ≥ 0.95, and $\:{\chi\:}^{2}$/df < 3.

##### Convergent validity

Convergent validity was assessed using standardized factor loadings (acceptable >0.50, strong >0.70 [76]), average variance extracted (AVE ≥ 0.50 [77]), and composite reliability (CR ≥ 0.70 [76]).

##### Discriminant validity

We also assessed discriminant validity, i.e., whether the absolute values of the correlations between the factors were low enough to consider them representing distinct subscales. According to the recommendations of Rönkkö and Cho [78], the CICFA(sys) method was used to evaluate discriminant validity. We calculated the 95% CIs of the estimated factor correlations for the latent factors in the model. Following Rönkkö and Cho’s classification system [78], we interpreted discriminant validity based on the upper limits (UL) of the confidence intervals: UL ≥ 1.00 indicated a severe problem, 0.90 ≤ UL < 1.00 a moderate problem, 0.80 ≤ UL < 0.90 a marginal problem, and UL < 0.80 no problem.

##### Reliability

Reliability was assessed using Cronbach’s alpha and McDonald’s omega, with values ≥ 0.90 considered excellent, ≥ 0.80 strong, and ≥ 0.70 acceptable [73]. This applied both to the NBIS-P-G subscales and the multi-item external measures used in the concurrent validity analysis (i.e., brand attitudes, allyship, and social change commitment).

##### Concurrent validity

To assess concurrent validity, Pearson’s r correlations were computed between the NBIS-P-G subscales and theoretically related constructs: brand attitudes, occupational prestige, career recommendation likelihood, and political solidarity. Interpretation of correlations considered empirical magnitude, theoretical coherence, and context [69, 79], with emphasis on whether the direction and size of the associations aligned with expectations based on the subscale content.

## Results

### Participant data

Data collection resulted in 1,245 responses. After excluding cases not meeting the inclusion criteria (*n* = 28), failing the commitment and attention checks (*n* = 164), or not passing the longstring check (*n* = 103), the final sample comprised 950 participants.

The final sample was nearly evenly split by gender, with 51.7% women and 48.3% men. Participants aged 50–65 represented the largest age group (31.4%; see Table 2). The highest proportion of respondents held higher education degrees (43.3%). Most participants were part of the labor force (74.8%). Overall, the demographic profile of the sample closely reflected the characteristics of the target population [80, 81].

**Table 2:** Characteristics of the sample

	Sample			Target population
	*n*	%		%
**Gender**				
Female	491	51.7		49.3
Male	459	48.3		50.7
**Age (years)**				
16–29	243	25.6		24.9
30–39	217	22.8		20.8
40–49	192	20.2		19.5
50–65	298	31.4		34.8
**Education level**				
Low	218	22.9		25.6
Mid	321	33.8		32.3
High	411	43.3		42.1
**Employment**				
Employed (incl. actively seeking work)	711	74.8		75.7
Education/training	113	11.9		11.5
Housemen/-wives	61	6.4		7.3
Pension	65	6.8		5.5

Note: *N* = 950; education level: highest qualification according to Mikrozensus, low = compulsory school / lower secondary level school (completion grade 9), mid = lower secondary level school (completion grade 10), high = university (of applied sciences) entry qualification / university degree

### Structural validity

Initial assessment of structural validity was conducted using CFA on the full sample (*N* = 950). The model, based on the hypothesized seven-factor structure with 42 indicators, did not meet commonly accepted cut-off criteria for adequate model fit (RMSEA [90% CI] = 0.061 [0.058, 0.063], SRMR = 0.053, CFI = 0.889, χ2/df = 2,654.95/798 = 3.33), indicating that the hypothesized structure required re-examination.

Therefore, and in accordance with guidelines for scale development and validation [69], we implemented a two-step approach to explore and confirm the latent structure. Following the N:p heuristic with a cases-per-variable ratio of 10:1 [82], we randomly split the sample, allocating 420 participants to exploratory factor analysis (EFA) and retaining the remaining 530 for subsequent confirmatory factor analysis (CFA).

#### Exploratory factor analysis

Bartlett’s test of sphericity was significant (*p* < 0.001), and the Kaiser-Meyer-Olkin measure verified sampling adequacy for EFA, with an overall KMO = 0.98 and all individual item KMO values exceeding 0.90, well above the acceptable limit of 0.50.

Given the factorability, multivariate non-normality, and expected correlated factors, EFA using principal axis factoring with Promax rotation was conducted to identify the dimensional structure of the 42-item NBIS-P-G [69, 83]. Convergence of multiple factor extraction criteria (parallel analysis, Kaiser criterion, optimal coordinates index, and Velicer’s MAP criterion) supported a 4-factor solution.

Items were systematically evaluated using three criteria to eliminate problematic cross-loadings and enhance factor structure clarity [83]: Hoffman’s index of complexity >2.5 (indicating high cross-loadings), communality < 0.40 (insufficient explained variance), and primary loadings < 0.40 (weak factor membership). Six items were removed based on multiple criterion violations or extreme single-criterion deficiencies: three items (22, 32, 41; see Table 1 for reference) violated both complexity and loading criteria, while items 38 and 40 demonstrated extreme complexity (>3.0) and item 37 showed insufficient communality (h² = 0.32). This approach retained 36 items (86% of original scale), removing only items with the most severe deficiencies while preserving content validity [83].

The refined 36-item, 4-factor solution demonstrated good model fit (RMSEA [90% CI] = 0.045 [0.040, 0.049], RMSR = 0.02, TLI = 0.951) and explained 60.1% of total variance (Table 3). Primary factor loadings ranged from 0.35 to 0.91, with 97% of items ≥ 0.40 and 81% ≥0.50. Cross-loadings were limited, with only 5 items showing secondary loadings >0.30. Item complexity was low (M = 1.42), and communalities ranged from 0.47 to 0.78 (M = 0.60), indicating adequate shared variance. Inter-factor correlations (0.41–0.75) were consistent with research documenting halo effects in brand perception [84].

**Table 3:** Factor loadings and communalities for the EFA of the adjusted NBIS-P-G

Items		Factors				
NBIS-P-G	NBIS	1	2	3	4	h^2^
fachkundig	Skilled	0.81				0.69
professionell	Professional	0.73				0.64
Brückenbauer*innen in der Kommunikation	Communicators	0.72				0.56
umfassende Ausbildung	Extensive Training	0.70				0.58
kompetent	Competent	0.69				0.66
ganzheitlicher Ansatz	Holistic Approach	0.65				0.64
interprofessionell	Interprofessional	0.64				0.64
ehrlich/Integrität	Honest/Integrity	0.63				0.58
ethisch	Ethical	0.62				0.50
achtsam und umsichtig	Intuitive/Thoughtful	0.62				0.57
verlässlich/zuverlässig	Reliable/Dependable	0.60	0.35			0.63
evidenzbasierte Praxis	Evidence Based Practice	0.56		0.33		0.58
Pflegewissenschaftler*innen	Researchers	0.53				0.53
reflektiert/kritisch denkend	Critical Thinkers	0.52				0.55
sachkundig/intelligent	Knowledgeable/Intelligent	0.49	0.30			0.61
interprofessionelle Partner*innen	Collaborators/Facilitators	0.46				0.57
fürsorglich/mitfühlend	Caring/Compassionate		0.91			0.78
verbringt die meiste Zeit mit Patient*innen	Spends Most Time With Patients		0.82			0.66
empathisch	Empathetic		0.80			0.72
genießt Vertrauen	Trusted		0.75			0.70
patientenorientiert	Patient Centered/Focused		0.74			0.76
umsorgend/bemutternd	Nurturing/Mothering		0.67			0.70
Fürsprecher*innen	Advocates		0.54	0.34		0.66
ärztliche Hilfskraft	Physician’s Assistant		0.52			0.55
talentiert/begabt	Talented		0.35			0.58
mächtig/Entscheidungsträger*innen	Powerful/Decision Makers			0.83		0.59
Führungspersonen	Leaders			0.81		0.62
einflussreich	Influential			0.68		0.56
höhere Bildungsabschlüsse	Advanced Degrees			0.53		0.50
von Gesellschaft/Gesundheitswesen wertgeschätzt	Valued by Society/Healthcare			0.51		0.50
eigenständig	Autonomous			0.47		0.47
kompetent im Umgang mit Technik	Technological			0.47		0.56
Gesundheitsexpert*innen	Health Experts		0.40	0.46		0.55
untergeordnet/dienend	Subservient				0.87	0.63
Berufskleidung vermittelt keine Professionalität	White Cap/Uniform				0.57	0.47
schwer von anderen zu unterscheiden	Hard to identify from others				0.47	0.58
**Proportion Variance (%)**		23.6	18.1	13.9	4.5	

Note: *N* = 420 (random split-sample for exploratory factor analysis); extraction method: principal axis factoring; rotation method: Promax; values represent standardized loadings (pattern matrix); loadings < 0.30 are suppressed for clarity; h² = communality; NBIS-P-G and NBIS item labels abbreviated for presentation; the four-factor solution accounted for 60.1% of the total variance

Based on analysis of item content, factor loading patterns, and alignment with the original English-language NBIS dimensions [35], the four factors were interpreted and labeled: *Professional Competence and Expertise* (Factor 1, 16 items, 23.6% variance), *Patient-Centered Care and Compassion* (Factor 2, 9 items, 18.1% variance), *Leadership and Influence* (Factor 3, 8 items, 13.9% variance), and *Professional Identity Challenges* (Factor 4, 3 items, 4.5% variance).

#### Confirmatory factor analysis

The 4-factor structure proposed by the EFA underwent confirmatory factor analysis using the robust maximum likelihood estimator in the independent split-sample (*N* = 530). The model demonstrated adequate fit (RMSEA [90% CI] = 0.045 [0.040, 0.049], SRMR = 0.054, CFI = 0.942, χ2/df = 1,009.44/588 = 1.72), representing substantial improvement over the initial 7-factor model and confirming adherence to the proposed 4-factor structure.

### Convergent validity

Convergent validity was evaluated based on standardized factor loadings, AVE, and CR (Table 4). All standardized factor loadings from the CFA model were statistically significant (*p* < 0.001). Standardized loadings ranged from 0.620 to 0.816, exceeding the commonly recommended minimum of 0.50. In total, 19 of 36 items demonstrated strong loadings above 0.70 on their respective factors.

The AVE values were 0.500 for Factor 1, 0.547 for Factor 2, 0.452 for Factor 3, and 0.492 for Factor 4. While Factors 1 and 2 met or exceeded the conventional 0.50 threshold, Factors 3 and 4 fell below, suggesting that the factors account for a more modest proportion of item variance.

Composite reliability was high for Factors 1 (CR = 0.942), 2 (CR = 0.917), and 3 (CR = 0.871), and acceptable for Factor 4 (CR = 0.739), with all values above the 0.70 threshold.

Following recommendations [85], we conducted a post-hoc assessment of convergent validity using 90% percentile bootstrap confidence intervals for AVE (1,000 samples) to account for sampling uncertainty. This approach tests whether AVE is significantly below the 0.50 threshold by examining whether the upper bound of the 90% CI exceeds 0.50. Three of four factors demonstrated convergent validity with 90% CIs of F1 [0.462–0.537], F2 [0.507–0.589], and F4 [0.434–0.546], while F3 (90% CI [0.415–0.491]) did not meet this criterion.

**Table 4:** Standardized factor loadings, AVE, and CR for the NBIS-P-G factors

Items		Factors			
NBIS-P-G	NBIS	1	2	3	4
evidenzbasierte Praxis	Evidence Based Practice	0.669			
interprofessionell	Interprofessional	**0.718**			
achtsam und umsichtig	Intuitive/Thoughtful	**0.720**			
Brückenbauer*innen in der Kommunikation	Communicators	0.678			
kompetent	Competent	**0.746**			
interprofessionelle Partner*innen	Collaborators/Facilitators	0.662			
reflektiert/kritisch denkend	Critical Thinkers	0.657			
professionell	Professional	**0.754**			
sachkundig/intelligent	Knowledgeable/Intelligent	**0.736**			
ehrlich/Integrität	Honest/Integrity	**0.730**			
ethisch	Ethical	**0.715**			
Pflegewissenschaftler*innen	Researchers	0.654			
ganzheitlicher Ansatz	Holistic Approach	**0.719**			
umfassende Ausbildung	Extensive Training	0.696			
verlässlich/zuverlässig	Reliable/Dependable	**0.709**			
fachkundig	Skilled	**0.746**			
talentiert/begabt	Talented		0.652		
verbringt die meiste Zeit mit Patient*innen	Spends Most Time With Patients		0.680		
genießt Vertrauen	Trusted		**0.783**		
empathisch	Empathetic		**0.732**		
fürsorglich/mitfühlend	Caring/Compassionate		**0.796**		
Fürsprecher*innen	Advocates		**0.791**		
patientenorientiert	Patient Centered/Focused		**0.816**		
ärztliche Hilfskraft	Physician’s Assistant		0.650		
umsorgend/bemutternd	Nurturing/Mothering		**0.737**		
eigenständig	Autonomous			0.668	
einflussreich	Influential			0.686	
mächtig/Entscheidungsträger*innen	Powerful/Decision Makers			0.678	
Führungspersonen	Leaders			**0.715**	
höhere Bildungsabschlüsse	Advanced Degrees			0.659	
kompetent im Umgang mit Technik	Technological			0.690	
Gesundheitsexpert*innen	Health Experts			0.657	
von Gesellschaft/Gesundheitswesen wertgeschätzt	Valued by Society/Healthcare			0.629	
schwer von anderen zu unterscheiden	Hard to identify from others				**0.710**
Berufskleidung vermittelt keine Professionalität	White Cap/Uniform				**0.759**
untergeordnet/dienend	Subservient				0.620
**AVE**		0.500	0.547	0.452	0.492
**CR**		0.942	0.917	0.871	0.739

Note: *N* = 530 (random split-sample for confirmatory factor analysis); NBIS-P-G and NBIS item labels abbreviated for presentation; Factor 1 = Professional Competence and Expertise, Factor 2 = Patient-Centered Care and Compassion, Factor 3 = Leadership and Influence, Factor 4 = Professional Identity Challenges; all loadings significant at *p* < 0.001; bold = loadings > 0.70; AVE = Average Variance Extracted; CR = Composite Reliability

### Discriminant validity

Discriminant validity was assessed using the CICFA(sys) method [78]. Table 5 reports the latent factor correlations from the CFA model along with their 95% confidence intervals (CIs). Comparing the upper limits (ULs) of these CIs to the CICFA(sys) classification system revealed no evidence of discriminant validity problems for correlations between Factors 1, 2, and 3 with Factor 4 (all ULs < 0.80).

The ULs of the correlations between Factors 1 and 3 (95% CI [0.747, 0.859]) and Factors 2 and 3 (95% CI [0.680, 0.820]) fell between 0.80 and 0.90. According to the classification system, this indicated marginal discriminant validity concerns, suggesting that the respective subscales can still be interpreted as representing distinct brand image dimensions [78].

**Table 5:** Latent factor correlations with 95% confidence intervals

	Factor 1	Factor 2	Factor 3	Factor 4
**Factor 1**: Professional Competence and Expertise	1.00			
**Factor 2**: Patient-Centered Care and Compassion	0.885 [0.844, 0.925]	1.00		
**Factor 3**: Leadership and Influence	0.803 [0.747, 0.859]	0.750 [0.680, 0.820]	1.00	
**Factor 4**: Professional Identity Challenges	0.358 [0.218, 0.498]	0.467 [0.332, 0.602]	0.690 [0.584, 0.796]	1.00

Note: *N* = 530 (random split-sample for confirmatory factor analysis); values in square brackets indicate the 95% confidence interval for each correlation; all correlations are significant at *p* < 0.001

The correlation between Factors 1 and 2 showed a 95% CI of [0.844, 0.925], placing the UL in the range of 0.90 to 1.00. This indicated a moderate discriminant validity concern, warranting closer scrutiny of the conceptual distinctiveness of these two factors [78].

None of the CIs reached or exceeded an upper limit of 1.00, and thus, no severe discriminant validity problems were identified [78].

### Reliability

Following the EFA and CFA conducted on separate random subsamples, subsequent reliability and concurrent validity analyses were performed using the full sample (*N* = 950) to ensure statistical power and generalizability. Internal consistency estimates are presented in Table 6.

For the *Professional Competence and Expertise* subscale, both Cronbach’s alpha and McDonald’s omega were 0.95, indicating excellent internal consistency. The *Patient-Centered Care and Compassion* subscale yielded a Cronbach’s alpha of 0.93 and a McDonald’s omega of 0.94, also reflecting excellent internal consistency.

**Table 6:** Internal consistency of NBIS-P-G subscales

NBIS-P-G Subscale	Cronbach’s α	McDonald’s ω
1. Professional Competence and Expertise	0.95	0.95
2. Patient-Centered Care and Compassion	0.93	0.94
3. Leadership and Influence	0.88	0.91
4. Professional Identity Challenges	0.74	0.74

Note: *N* = 950; Subsample reliability analyses confirmed stable estimates across EFA (*N* = 420) and CFA (*N* = 530) subsamples (EFA/CFA): Factor 1 (α = 0.94/0.95, ω = 0.95/0.96), Factor 2 (α = 0.92/0.94, ω = 0.94/0.95), Factor 3 (α = 0.87/0.89, ω = 0.89/0.91), Factor 4 (α = 0.74/0.75, ω = 0.75/0.75)

The *Leadership and Influence* subscale showed strong internal consistency, with Cronbach’s alpha of 0.88 and McDonald’s omega of 0.91. For the *Professional Identity Challenges* subscale, Cronbach’s alpha and McDonald’s omega were both 0.74, suggesting acceptable internal consistence.

In addition, the multi-item external measures used in the concurrent validity analysis demonstrated strong internal consistency in the present sample, with Cronbach’s alpha and McDonald’s omega values as follows: brand attitudes (α = 0.89, ω = 0.91), allyship (α = 0.86, ω = 0.87), and social change commitment (α = 0.89, ω = 0.89).

### Concurrent validity

Table 7 presents Pearson correlations of the NBIS-P-G subscales with brand attitudes, occupational prestige, career recommendation likelihood, and political solidarity (allyship and social change commitment).

*Professional Competence and Expertise* showed moderate positive correlations with brand attitudes (*r* = 0.48) and occupational prestige (*r* = 0.32), as well as a smaller correlation with career recommendation likelihood (*r* = 0.21). Associations with political solidarity were moderate (allyship: *r* = 0.41, social change commitment: *r* = 0.32) (all *p* < 0.001).

*Patient-Centered Care and Compassion* demonstrated a similar pattern: moderate correlations with brand attitudes (*r* = 0.48) and occupational prestige (*r* = 0.33), small correlation with career recommendation likelihood (*r* = 0.25), and moderate correlations with political solidarity (allyship: *r* = 0.40, social change commitment: *r* = 0.30) (all *p* < 0.001).

**Table 7:** Pearson correlations between scales: concurrent validity analysis

NBIS-P-G Subscale	BRA	BAS	CRL	PSM-A	PSM-SCC
1. Professional Competence and Expertise	0.48^***^	0.32^***^	0.21^***^	0.41^***^	0.32^***^
2. Patient-Centered Care and Compassion	0.48^***^	0.33^***^	0.25^***^	0.40^***^	0.30^***^
3. Leadership and Influence	0.44^***^	0.38^***^	0.34^***^	0.26^***^	0.08^*^
4. Professional Identity Challenges	0.22^***^	0.21^***^	0.23^***^	0.05	-0.11^**^

Note: *N* = 950; BRA = Brand Attitudes; BAS = Occupational Prestige; CRL = Career Recommendation Likelihood; PSM-A = Allyship; PSM-SCC = Social Change Commitment; ^*^ *p* < 0.05, ^**^ *p* < 0.01, ^***^ *p* < 0.001, Holm-adjusted p-values for multiple comparisons

*Leadership and Influence* showed a slightly lower correlation with brand attitudes (*r* = 0.44, *p* < 0.001), but stronger associations with occupational prestige (*r* = 0.38, *p* < 0.001) and career recommendation likelihood (*r* = 0.34, *p* < 0.001). Correlations with political solidarity were small (allyship: *r* = 0.26, *p* < 0.001; social change commitment: *r* = 0.08, *p* < 0.05).

*Professional Identity Challenges* correlated weakly with brand attitudes (*r* = 0.22, *p* < 0.001), occupational prestige (*r* = 0.21, *p* < 0.001), and career recommendation likelihood (*r* = 0.23, *p* < 0.001). No significant association was found with allyship (*p* = 0.20), and a small negative correlation emerged with social change commitment (*r* = -0.11, *p* < 0.01).

Overall, these patterns support the concurrent validity of the NBIS-P-G subscales. The first two subscales consistently aligned with favorable brand perceptions and solidarity-related attitudes. *Leadership and Influence* also showed meaningful, primarily occupationally anchored associations. The weaker or absent associations of *Professional Identity Challenges* will be explored further in the discussion.

## Discussion

The aim of this study was to translate and validate the Nursing Brand Image Scale (NBIS) for use in the German general population. This represents the first validation study of the German NBIS and thus extends current knowledge in this research field.

First, our findings suggest a revised factorial structure for the NBIS-P-G, diverging from the original seven-factor models proposed by Godsey et al. [35] (U.S.) and Zhou et al. [46] (China). The initial CFA failed to meet established fit thresholds, prompting an exploratory-confirmatory approach in line with recommended scale adaptation procedures [69]. The EFA revealed a four-factor structure that better represented the latent dimensionality within the German context. This solution was strongly supported both statistically (e.g., RMSEA = 0.045, RMSR = 0.02, TLI = 0.951; explained variance = 60.1%) and conceptually. Notably, it retained 86% of the original items, striking a balance between content validity and psychometric rigor [83]. The resulting factors – *Professional Competence and Expertise*, *Patient-Centered Care and Compassion*, *Leadership and Influence*, and *Professional Identity Challenges* – were confirmed via CFA in the validation subsample, showing acceptable fit (RMSEA = 0.045, SRMR = 0.054, CFI = 0.942), and representing a clear improvement over the original structure.

Conceptually, the more parsimonious structure potentially reflects both cross-cultural differences in perceptions of nursing [5, 13, 26] and population differences between the general public and nurse samples. The NBIS-P-G was applied to the general public, whereas the original NBIS was validated in nurses [35, 46]. Prior studies show that self- and other-perceptions of nursing diverge [29, 30], and our results align with this pattern. Branding research provides further explanation: brand images are associative knowledge structures [38, 40], shaped by experience and learning. Experts typically hold more elaborate and differentiated brand associations than non-experts, who tend to form more holistic, less granular impressions [86]. Thus, it is plausible that nurses, as insiders, possess a more multidimensional image of their profession than the general public.

Importantly, the NBIS-P-G’s four-factor solution shows partial parallels with U.S. and Chinese public versions (NBIS-P-US/C) [26]. Two NBIS-P-G factors, *Leadership and Influence* and *Professional Identity Challenges*, appear conceptually related to the NBIS-P-US/C’s *Leader Roles/Traits* and *Lack of Authority/Identity* factors. Both pairs capture similar domains: the former includes autonomy, influence, and advanced qualifications, though the NBIS-P-G emphasizes authority and decision-making more narrowly. The latter pair appears broadly consistent in thematic content reflecting stereotypes, subordination and professional ambiguity.

In contrast, the NBIS-P-G’s *Professional Competence and Expertise* and *Patient-Centered Care and Compassion* suggest a more differentiated structure than the two remaining NBIS-P-US/C factors, which combine technical and relational attributes. This distinction resonates with a recent review of nursing image [5], separating professional knowledge, technical/cognitive/organizational professional skills, and competence-related professional spirits aspects (honesty/integrity, ethics) from the interpersonal dimensions of professional skills and relational/affective aspects of professional spirit (e.g., empathy, compassion, advocacy).

Regarding our *Patient-Centered Care and Compassion* factor specifically, recent reviews reveal varied conceptualizations of patient-centered care, with some frameworks including empathy and compassion elements as one defining element among others, while alternative approaches position empathy and compassion attributes as facilitators rather than core components of patient-centered care [87, 88]. Our empirical findings suggest that the German public perceives these aspects as closely related elements of nursing practice. Notably, “ärztliche Hilfskraft” loaded on this factor, contrasting with its association with professional identity challenges in nurse samples [35, 46] but paralleling its allocation to “Caregiver virtues/attributes” in public samples [26]. This differential allocation may reflect divergent public versus professional perceptions or methodological considerations related to item interpretation, warranting further investigation.

Returning to the broader structural differentiation observed in our NBIS-P-G factor solution, the empirical separation between competence and relational dimensions also resonates with the Stereotype Content Model [89], which distinguishes “competence” (i.e., perceived capability and efficacy) and “warmth” (i.e., perceived kindness and relational orientation) as core social and brand perception dimensions [90]. While no direct equivalence is implied, *Professional Competence and Expertise* shares features of the competence dimension, and *Patient-Centered Care and Compassion* reflects relational warmth.

Additionally, contextual factors may help explain the competence-compassion distinction observed for the NBIS-P-G. In Germany, sustained COVID-19 media coverage drew public attention to nursing [33], highlighting both technical expertise in high-stakes settings and emotional labor. Beyond this, the distinction may reflect a dual-prototype structure in public cognition. Prototype theory [91, 92] posits that categories are mentally represented via prototypical exemplars; in Germany, two basic-level prototypes [91, 92] of nursing may be salient: the “hospital nurse” and the “geriatric nurse”. These prototypes may have been reinforced by Germany’s historically differentiated vocational training pathways, only unified in 2020 by the German Nursing Professions Act [93], and distinct practice settings. If such a structure is present, the “hospital nurse” prototype would be more strongly associated with technical competencies, while the “geriatric nurse” prototype would be more strongly associated with relational continuity and compassionate caregiving. When evaluating “nursing” in general, respondents may draw upon both prototypes simultaneously with different weights, yielding the observed competence-compassion distinction. Together, pandemic framing and dual-prototype structure may have contributed to the sharper distinction in the German public’s nursing image.

Taken together, these findings support the structural validity of the NBIS-P-G. The four-factor model is psychometrically sound and theoretically grounded, making it suitable for capturing public perceptions of nursing in Germany.

As part of model refinement, six items were removed due to psychometric deficiencies. These warrant brief discussion. “Gesundheitsdienstleister*innen” (Health Care Providers) showed high factorial complexity, potentially due to its abstract, umbrella like use in public discourse. Items such as “unverzichtbare Mitglieder des Gesundheitsteams” (Essential Members of Healthcare Team), “vielfältige berufliche Möglichkeiten” (Diverse Career Options), and “Lehrpersonen/Ausbilder*innen” (Teacher/Educator) combined high factorial complexity with weak loadings and may represent aspirational or less visible image facets [13, 35], limiting their salience [5]. “Aufgabenfixiert” (Task Oriented) may have been semantically ambiguous, being interpreted positively (efficient) or negatively (rigid), leading to unstable responses. “Weiblich” (Female) showed low communality. While it loaded on *Professional Identity Challenges*, the association was too weak for retention. As noted in recent reviews [5, 13], nursing remains gender stereotyped, though these perceptions are contested. In a polarized gender discourse [94], “weiblich” may be seen by some as neutral, by others as stereotypical or problematic. This semantic ambiguity may have disrupted its coherence with other items. While these exclusions improved factorial clarity, they may have narrowed coverage of contested or emerging image facets. Future research should consider reintroducing these domains with refined indicators as public perceptions of nursing evolve.

Second, the results generally support convergent validity. Three of the four factors – *Professional Competence and Expertise*, *Patient-Centered Care and Compassion*, and *Professional Identity Challenges* – showed acceptable standardized factor loadings, strong CR, and exceeded AVE thresholds or showed upper bootstrap CI bounds above 0.50. *Leadership and Influence* presents a more nuanced picture. While its items’ factor loadings and CR meet standard criteria, AVE (0.452; 90% CI [0.415; 0.491]) falls below the 0.50 threshold, echoing similar findings in the Chinese NBIS [46]. While individual factor loadings are acceptable (0.629–0.715), limited shared variance suggests variation in public understanding of nursing leadership. Hence, this factor may benefit from targeted item refinement to strengthen convergent validity.

Third, factor correlations supported discriminant validity overall. All factor correlations remained below the critical threshold [78], with no confidence intervals including 1.00 – indicating that the four NBIS-P-G dimensions are statistically distinguishable. *Professional Identity Challenges* showed the clearest discriminant separation (all correlations < 0.80), while *Leadership and Influence* showed moderately elevated correlations with *Professional Competence and Expertise* (upper CI: 0.859) and *Patient-Centered Care and Compassion* (upper CI: 0.820), falling within the “marginal concern” range without threatening empirical distinctiveness [78].

The highest correlation between *Professional Competence and Expertise* and *Patient-Centered Care and Compassion* (*r* = 0.885, 95% CI [0.844–0.925]) falls into the “moderate concern” range based on its upper CI bound [78]. However, empirical distinctiveness remains acceptable with the lower CI bound within the “minor concern” range [85]. Conceptually, these factors capture distinct domains – technical/cognitive versus relational/emotional qualities – reflecting the validated competence-warmth framework [89, 90]. Moreover, a high correlation can be theoretically expected given that competence and compassion associations likely represent distinct yet closely linked and frequently co-activated clusters in public discourse and personal experience [95]. Branding theory similarly describes such halo effects between favorable traits [84]. Future research should nevertheless refine items to sharpen distinctions between these constructs.

Fourth, the internal consistency reliability of the NBIS-P-G subscales was satisfactory. All four subscales demonstrated acceptable to high reliability, with both Cronbach’s alpha and McDonald’s omega exceeding the conventional threshold of 0.70 (α, ω ≥ 0.70).

Fifth, concurrent validity was supported by theory-congruent associations with external constructs. *Professional Competence and Expertise* and *Patient-Centered Care and Compassion* correlated moderately with brand attitudes, occupational prestige, and political solidarity.

*Leadership and Influence* showed stronger links to occupational prestige and career recommendation, but weaker ties to political solidarity – possibly reflecting disconnect between public recognition of leadership traits and solidarity-driven engagement.

*Professional Identity Challenges* showed weak but positive correlations with brand attitudes and career recommendation. This suggests that image concerns do not preclude a generally favorable evaluation of nursing. However, its absent or slightly negative correlations with political solidarity invite further interpretation.

One explanation, drawn from Social Identity Theory [96], is that individuals high in political solidarity may identify with nursing as a valued group and strive to preserve a positively distinctive image – making them less likely to endorse deficit-framed items. A second interpretation, based on Critical Consciousness Theory [97], posits that those high in political solidarity may acknowledge structural and representational challenges but avoid framing them in ways that could undermine political efficacy or collective agency. Third, individuals who do endorse such items might be critically aware of structural issues, but adopt a more observational stance that lacks the value-driven engagement reflected in political solidarity measures [97].

Sixth, with respect to cross-national (non)invariance, our German public data extend the NBIS evidence base. Whereas the original U.S. nurse sample supported a seven-factor structure [35], and Zhou et al. [26] identified a four-factor solution that was configural- and metric-invariant across U.S. and Chinese public samples, the NBIS-P-G likewise yielded four factors. This convergence in the number of factors suggests potential dimensional invariance across public (non-nurse) populations [98, 99]. However, the factor structure in the German sample diverges conceptually – for instance, showing a sharper split between competence- and care-related attributes – indicating potential configural noninvariance. Since configural invariance is a prerequisite for metric, scalar and error invariance, our results raise doubts about the scale’s suitability for direct cross-national comparisons in its current form. Formal testing of measurement invariance, particularly via multigroup CFA, should be undertaken to confirm these patterns. If partial measurement invariance is found, only items meeting criteria for strong invariance should be used in cross-national composite scores [99]. As more comparative data become available, the NBIS may benefit from revision to support robust cross-national applications.

### Limitations and paths for future research

Some limitations of this study should be acknowledged. First, its cross-sectional design precluded the assessment of the NBIS-P-G’s reproducibility. Future research should address this by evaluating both test-retest reliability (relative measurement error) and agreement (absolute measurement error) [100, 101]. As part of examining longitudinal validity, future studies should also assess responsiveness, i.e., the instrument’s ability to detect meaningful changes in the nursing brand image over time [101].

Second, although a large quota-based sample – representative in terms of age, gender, education, employment, and region – supports a degree of generalizability, the use of non-probability online panel sampling introduces potential bias and limits external validity [52, 53, 64]. Because participants were compensated, economic self-selection may have influenced the panel composition [52]. To mitigate this, quotas based on employment and education – serving as socioeconomic proxies – were applied, and incentives were deliberately modest. Additionally, the online format may have led to overrepresentation of technology-savvy individuals. The age range in this study was restricted to 16–65 years, excluding both younger adolescents and older adults – the latter being among the most intensive users of healthcare services [102, 103]. Some prior research suggests that (1) older adults may hold more traditional, care-oriented views of nursing [104, 105]; (2) may have a more limited understanding of nursing roles, responsibilities, and capabilities [104]; and (3) are more likely to have direct contact with nursing staff due to higher rates of healthcare utilization [102, 103] offering more opportunities to learn about various aspects of the profession. These factors could influence how nursing attributes are perceived and interpreted, potentially affecting the factor structure – for example, making it less differentiated due to conceptual overlap, or more nuanced due to firsthand experience. However, other evidence is less conclusive: Zhou et al. [26] found that age did not significantly influence public perceptions of nursing’s brand image in either China or the US. Younger adolescents may also hold distinct views; for instance, Cirik et al. [106] found that children viewed nurses more positively than their parents. Broader age inclusion in future studies may therefore help assess the stability and generalizability of the instrument across the lifespan.

A third potential limitation concerns the AI-assisted forward and back translation. While AI can expedite cross-language instrument work when paired with expert validation, it also introduces risks that require cultural sensitivity and transparent safeguards [48]. We followed recent guidance on AI-supported questionnaire translation [48], leveraging the relatively low linguistic distance and high-resource status of English-German [48, 107] and using informed prompting (context, target group, quality criteria) [108–110], then embedding AI outputs in a structured human workflow (harmonization, expert review, pretesting, and verification with an NBIS author) [48, 51, 108]. Despite these safeguards, we identified and addressed instances such as occasional lexical choices that missed professional nuance; loss of distinction (different English items mapped to the same German term) and loss of coherence (inconsistent terms within a semantic field); and cases of technical register where lay wording would be preferable. While these issues were resolved in the final instrument, subtle nuance errors cannot be fully ruled out as influencing the factor structure, internal consistency, or measurement invariance across language versions. Future work might complement expert review with cognitive interviewing focused on nuance and structured post-editing using MQM criteria (accuracy, fluency, terminology, style) to systematize corrections [110, 111].

Fourth, the use of self-report measures introduces a general risk of common method variance. According to Spector et al.’s categorization of method variance sources [112], the attitudinal nature of the construct under investigation makes social desirability a particularly likely source of common method variance in this context. Future research could address this by applying the directly measured latent method factor technique [113] to account for social desirability or other directly measurable sources of common method variance. Alternatively, the CFA marker technique may be appropriate for capturing method variance that is neither anticipated nor directly measurable [114].

Fifth, two external reference measures used in the concurrent validity analysis – occupational prestige and career recommendation likelihood – were assessed using single-item instruments to ensure survey efficiency. While such measures are considered acceptable for narrow, clearly defined constructs, they do not allow for internal consistency assessment [115]. Established procedures for validating single-item instruments (e.g., test-retest) [115] were beyond the scope of this study. Future studies may benefit from using multi-item measures for these constructs to allow for more comprehensive reliability and validity testing.

Sixth, the use of mandatory responses in the survey may be considered a limitation. This approach can elicit reactance in respondents, potentially affecting data quality [68, 116]. However, such effects are more likely to occur with sensitive or personal topics, which were not the focus of this study. Therefore, the risk of compromised data quality due to this design choice is likely limited in this context.

Seventh, the adequacy of the sample size merits consideration. The a-priori calculation – based on a planned seven-factor CFA – was satisfied. However, as the initial model did not achieve acceptable fit, an exploratory-confirmatory approach was adopted, requiring a split of the total sample for EFA and CFA. To support the EFA, we deliberately allocated a subsample of 420 participants, applying the commonly recommended 10:1 ratio of cases per variable for the 42-item scale [82]. Following this, the remaining subsample (*N* = 530) was used for the CFA. A post hoc evaluation using Koran’s simulation-based heuristic [70] indicates that, given the final four-factor model with at least three indicators per factor and an average standardized loading of 0.71, a sample of approximately 350 would have sufficed. While the split-sample strategy was not part of the original study design, these considerations suggest that the final sample size was appropriate for the analytical procedures employed.

Future research should move beyond addressing the general study limitations and continue to optimize the instrument’s psychometric properties. First, the modified four-factor structure of the NBIS-P-G needs replication in independent German samples to establish its stability and guard against capitalizing on incidental characteristics of the present data set [69]. Second, although factorial clarity improved by excluding weak items, these exclusions may have narrowed thematic coverage of certain emerging or contested domains of the nursing brand image. New indicators could be introduced to recapture those facets while maintaining factorial clarity. Third, the marginal convergent validity of the *Leadership and Influence* factor suggests that targeted item revision may be beneficial. Refining item wording, adding more precisely targeted indicators, or replacing underperforming items could help strengthen factor convergence. Similarly, discriminant validity between *Professional Competence and Expertise* and *Patient-Centered Care and Compassion* could be improved by re-articulating items to emphasize each construct’s unique focus. Future research should also extend cross-national invariance testing by including public samples from Germany and other cultural contexts to assess the measurement invariance of the NBIS across a broader international spectrum. If full invariance is not achieved, studies could identify a subset of items meeting strong invariance criteria. Based on this, a core item set could be developed and validated, or the NBIS revised to enable meaningful international comparisons of the nursing brand image.

Another avenue for future research involves applying the validated NBIS-P-G to investigate the public brand image of nursing in Germany. This could involve using the instrument to provide a snapshot of how the German public perceives nursing by assessing and comparing NBIS-P-G subscale scores and individual items. Amidst ongoing debates about workforce shortages, professional roles, and healthcare system resilience, such findings could inform concrete priorities for branding strategies in the German context. This approach would enable evaluation of whether branding initiatives such as “All Nurses Are Leaders” [117] warrant prioritization in Germany, and identification of context-specific brand assets – potentially audience-specific – that require updating or strengthening. Regarding the latter, it would be particularly valuable to use the NBIS-P-G to examine heterogeneity in public perceptions of nursing in Germany. This could be accomplished by comparing pre-specified subgroups or through latent class analysis to identify distinct population segments with varying brand perceptions. Additionally, employing the NBIS-P-G longitudinally – to track changes over time or to assess effects of branding initiatives – would offer valuable insights into how the nursing brand image evolves and responds to branding efforts.

## Conclusions

This study provides the first German translation and validation of the Nursing Brand Image Scale for the public (NBIS-P-G). Using a large, representative quota-based sample (*N* = 950), a four-factor structure emerged – *Professional Competence and Expertise*, *Patient-Centered Care and Compassion*, *Leadership and Influence*, and *Professional Identity Challenges* – differing from previous validations. The NBIS-P-G demonstrated overall sound psychometric properties, including internal consistency and acceptable convergent, discriminant, and concurrent validity, with minor limitations particularly in convergent validity for the leadership factor. Despite limitations related to cross-sectional design, sampling, and certain aspects of validity requiring further refinement, the NBIS-P-G represents a valuable starting point for assessing and monitoring public perceptions of nursing in Germany. Future research should verify measurement invariance for robust cross-national comparisons and examine longitudinal changes to inform effective branding initiatives supporting nursing workforce sustainability on a global scale.

## Data Availability

The datasets used and/or analyzed during the current study are available from the corresponding author on reasonable request.
